# Genomic Sequences of Epizootic Hemorrhagic Disease Viruses Isolated from Florida White-Tailed Deer

**DOI:** 10.1128/genomeA.01174-17

**Published:** 2017-10-26

**Authors:** Kuttichantran Subramaniam, John A. Lednicky, Julia Loeb, Katherine A. Sayler, Samantha M. Wisely, Thomas B. Waltzek

**Affiliations:** a Department of Infectious Diseases and Immunology, College of Veterinary Medicine, University of Florida, Gainesville, Florida, USA; b Department of Environmental and Global Health, College of Public Health and Health Professions, University of Florida, Gainesville, Florida, USA; c Emerging Pathogens Institute, University of Florida, Gainesville, Florida, USA; d Department of Wildlife Ecology and Conservation, University of Florida, Gainesville, Florida, USA

## Abstract

*Epizootic hemorrhagic disease virus* (EHDV) serotypes 1 and 2 were isolated from Florida white-tailed deer in 2015 and 2016, respectively, and their genomes were completely sequenced. To our knowledge, these are the first full genome sequences for EHDV-1 and -2 from Florida.

## GENOME ANNOUNCEMENT

*Epizootic hemorrhagic disease virus* (EHDV) is a nonenveloped double-stranded RNA virus within the family *Reoviridae*, genus *Orbivirus* (1, 2). EHDV is one of the causative agents of hemorrhagic disease in ruminants and is the most economically important viral pathogens of white-tailed deer (*Odocoileus virginianus*) in the United States (3). Hematophagous flies of the genus *Culicoides* vector the virus, and deer infected with EHDV typically exhibit some or all of the following clinical signs: fever, anorexia, respiratory distress, and swelling of tissues of the head (e.g., periorbital region and tongue) and neck (3). Seven EHDV serotypes have been characterized worldwide, and of these, EHDV-1, -2, and -6 have been reported in white-tailed deer in the United States (2, 3).

During health assessments of deer suspected of having hemorrhagic disease performed by researchers from the University of Florida Cervidae Health Research Initiative, blood and spleen specimens were collected from farmed Florida white-tailed deer in 2015 (animal no. OV202) and 2016 (OV215), respectively. After EHDV viral RNA (vRNA) was detected in the blood (OV202) and spleen (OV215) specimens by reverse transcription-PCR (RT-PCR) (4), aliquots of whole blood and homogenized spleen were inoculated onto Vero E6 (*Cercopithecus aethiops* [African green monkey] kidney, ATCC CRL 1586) cells grown as monolayers at 37°C. After cytopathic effect became apparent, vRNA was extracted from virions in spent medium using a QIAamp viral RNA minikit (Qiagen), and a cDNA library was prepared using a NEBNext Ultra RNA library prep kit. Sequencing was performed using a version 3 600-cycle kit on a MiSeq platform (Illumina). *De novo* assembly of the paired-end reads was performed in SPAdes (5), recovering all 10 EHDV segments for both isolates. Gaps in segments or regions of low coverage were amplified by PCR followed by Sanger sequencing. Nucleotide sequences of the outer coat protein (VP2) from the two Florida isolates were aligned in MAFFT 5.8 (6) to EHDV-1, -2, and -6 orthologous sequences retrieved from the GenBank database. A maximum likelihood (ML) analysis was performed in IQ-TREE with default settings and 1,000 nonparametric bootstraps (7). The ML tree supported the isolates from OV202 and OV215 as EHDV-1 and -2, respectively. BLASTN analyses of the VP2 segments from OV202 and OV215 displayed highest nucleotide identity to an EHDV-1 (98% 2,860/2,916 bp; GenBank accession no. KU140746) and an EHDV-2 (99% 2,938/2,949 bp; GenBank accession no. KU140727) isolated from Missouri white-tailed deer in 2006 and 2012, respectively.

To date, these are the first cases of EHDV in white-tailed deer in Florida for which complete genome sequences have been determined. Future molecular epidemiological studies across a greater number of isolates are needed to determine whether EHDV strains circulating in white-tailed deer in Florida are unique or related to other regional strains.

### Accession number(s).

The genome sequences for EHDV-1 isolated from OV202 and EHDV-2 isolated from OV215 have been deposited in GenBank under accession no. MF688826 to MF688835 and MF688816 to MF688825, respectively.
